# PKH^high^/CD133+/CD24− Renal Stem-Like Cells Isolated from Human Nephrospheres Exhibit In Vitro Multipotency

**DOI:** 10.3390/cells9081805

**Published:** 2020-07-29

**Authors:** Silvia Bombelli, Chiara Meregalli, Chiara Grasselli, Maddalena M. Bolognesi, Antonino Bruno, Stefano Eriani, Barbara Torsello, Sofia De Marco, Davide P. Bernasconi, Nicola Zucchini, Paolo Mazzola, Cristina Bianchi, Marco Grasso, Adriana Albini, Giorgio Cattoretti, Roberto A. Perego

**Affiliations:** 1 School of Medicine and Surgery, Milano-Bicocca University, Via Cadore 48, 20900 Monza, Italy; c.meregalli4@campus.unimib.it (C.M.); c.grasselli@campus.unimib.it (C.G.); maddalena.bolognesi@unimib.it (M.M.B.); s.eriani@campus.unimib.it (S.E.); barbara.torsello@unimib.it (B.T.); s.demarco8@campus.unimib.it (S.D.M.); davide.bernasconi@unimib.it (D.P.B.); paolo.mazzola@unimib.it (P.M.); cristina.bianchi@unimib.it (C.B.); albini.adriana@gmail.com (A.A.); giorgio.cattoretti@unimib.it (G.C.); 2 IRCCS MultiMedica, 20138 Milan, Italy; antonino.bruno@multimedica.it; 3 Pathology Unit, ASST Monza, San Gerardo Hospital Via G.B. Pergolesi 33, 20900 Monza, Italy; n.zucchini@asst-monza.it; 4 Geriatric Unit, ASST Monza, San Gerardo Hospital Via G.B. Pergolesi 33, 20900 Monza, Italy; 5 Urology Unit, ASST Monza, San Gerardo Hospital Via G.B. Pergolesi 33, 20900 Monza, Italy; m.grasso@asst-monza.it

**Keywords:** human adult stem cell, kidney, endothelium, scaffold, nephrosphere, multipotency

## Abstract

The mechanism upon which human kidneys undergo regeneration is debated, though different lineage-tracing mouse models have tried to explain the cellular types and the mechanisms involved. Different sources of human renal progenitors have been proposed, but it is difficult to argue whether these populations have the same capacities that have been described in mice. Using the nephrosphere (NS) model, we isolated the quiescent population of adult human renal stem-like PKH^high^/CD133+/CD24− cells (RSC). The aim of this study was to deepen the RSC in vitro multipotency capacity. RSC, not expressing endothelial markers, generated secondary nephrospheres containing CD31+/vWf+ cells and cytokeratin positive cells, indicating the coexistence of endothelial and epithelial commitment. RSC cultured on decellularized human renal scaffolds generated endothelial structures together with the proximal and distal tubular structures. CD31+ endothelial committed progenitors sorted from nephrospheres generated spheroids with endothelial-like sprouts in Matrigel. We also demonstrated the double commitment toward endothelial and epithelial lineages of single RSC. The ability of the plastic RSC population to recapitulate the development of tubular epithelial and endothelial renal lineages makes these cells a good tool for the creation of organoids with translational relevance for studying the parenchymal and endothelial cell interactions and developing new therapeutic strategies.

## 1. Introduction

Neonephrogenesis does not occur in the adult human kidney, but the kidney retains some regenerative potential to replace the loss of cells during physiological processes, in which about 70,000 cells from different nephron segment are lost every hour and excreted with urine [1]. Even after acute kidney injury (AKI), regeneration is present, and AKI is considered reversible as documented by the recovery of urine production and the biomarkers of renal function [2]. Lineage-tracing studies in mice have helped clarify the mechanisms underlying the recovery after renal injury. Humphreys and colleagues [3], demonstrated that after AKI all new epithelial cells originate from within the renal epithelium itself and not from extrarenal sources. Some years later, Rinkevich and colleagues, in a “rainbow” mouse model, demonstrated the formation of new tubular cells via clonal expansion of specific tubular cell subpopulation in a segment-restricted manner [4]. It has long been known that in the kidney some tubular cells can undergo hypertrophy and some hyperplasia [5]. However, it remained to be determined whether any differentiated tubular cell under specific conditions is capable to give rise to dedifferentiation and clonal proliferation of tubular cells or if only a specific predetermined fraction of cells (stem/progenitor cells) is endowed with this potential within the nephron [6,7]. Lazzeri and colleagues [8] identified and characterized the tubular cells that undergo the endocycle-mediated hypertrophy and the small subset of Pax2+ tubular progenitor cells that have proliferative capacity in conditional Pax8/FUCCI2aR mice. With their paper they demonstrated in mice that the renal functional recovery upon AKI involves both tubular cell hypertrophy via endocycle and a limited regeneration driven by progenitors. They also observed the presence of endocycling cells as a dominant tubular epithelial response in human kidney biopsies from patients with chronic kidney disease after AKI [8]. However, the specific cell phenotype eventually responsible for tubular regeneration in human kidneys is still debated. In fact, different sources of multipotent renal progenitors have been described in different portions of the nephron, mainly based on CD133 expression, alone [9,10] or coupled with CD24 marker [11,12]. Since lineage tracing in humans is not applicable, it is difficult to argue whether these populations are capable of the same regenerative abilities described in mice and whether their phenotype is fixed or inducible [13].

In this scenario, we contributed to the debate by isolating a human cell population with stem features using the sphere-forming functional approach [14] that it has been recently described as a good model able to entails a genetic program that recapitulates renal development [15]. We identified the quiescent PKH26-retaining human renal stem-like cells (PKH^high^) isolated from clonal NS and demonstrated, in two dimensional (2D) culture, their ability to self-renew and differentiate into tubular and podocytic lineages as well as endothelial cells14. We also evidenced that 70% of PKH^high^ cells have the PKH^high^/CD133+/CD24− phenotype associated with stem-like properties [14]. Recently, we showed that our NS cells are able to repopulate tubular and endothelial structures, when cultured on decellularized human renal scaffolds [16].

In order to better understand whether, among the NS cells, the PKH^high^/CD133+/CD24− adult human renal stem-like cells (RSC) are the cellular players that drive repopulation and multipotency, the aim of this paper was to investigate in depth the in vitro multipotency of this cell subpopulation using different in vitro tests. In addition, aiming to give more insight on the cellular and molecular basis of renal regeneration, we also studied the in vitro multipotency capacity of RSC at the single cell level.

## 2. Materials and Methods

### 2.1. Tissues

Normal kidney tissue was obtained from 20 patients (male 14, female 6; median age 70 years, range 38–88 years) following nephrectomy for renal tumors. Discarded normal tissues far away from the tumor and exceeding the diagnostic needs were collected, de-identified and processed. The tissue was collected from the healthy region of the kidney without any presence of cancer and judged as normal by experienced pathologists. The procedures were approved (No 1532, 17 November 2011) by the institutional local Ethical Committee “Comitato Etico Azienda Ospedaliera San Gerardo” and patient informed consent was obtained. All procedures were performed in accordance with the Declaration of Helsinki, the relevant guidelines and regulations. NS cultures were established from fresh renal tissue samples. Frozen pieces of renal tissue, comprising cortex, medulla and papilla were stored at −80 °C until use.

### 2.2. Nephrosphere Cultures

The preparation of suspension of single cells from renal tissue [17] and the establishment of NS cultures [14] were performed as previously described. Cells before NS culture were stained with PKH26 lipophilic dye as described [14]. The floating NS grown in non-adherent conditions in dishes coated with poly Hema (Sigma-Aldrich, St.Louis, MO, USA) were collected for use after 10–12 days. NS cells were dissociated enzymatically for 5 min in TrypLE Express (Life Technologies, Waltham, MA, USA) and then mechanically by repetitive pipette syringing to generate a single cell suspension [14].

### 2.3. Immunofluorescence and FACS Analysis

Cytospin for immunofluorescence (IF) staining were prepared using cells after dissociation of NS or after sorting of specific cell subpopulations from dissociated NS. The slides were prepared spinning 5000 cells at 800× *g* for 15 min on Heraeus Multifuge 3S+ centrifuge (Thermo Scientific, Waltham, MA, USA). IF was performed as described [18] using the rabbit anti-von Willebrand factor (vWf, DAKO, Copenhagen, Denmark, 1:2000), mouse monoclonal anti-cytokeratin 8.18 (CK 8.18, clone 5D3, Thermo Fisher Scientific, Waltham, MA, USA, 1:50) and mouse monoclonal anti-CD31 (clone JC70A, DAKO, Copenhagen, Denmark, 1:25) primary antibodies and Alexa Fluor 488 conjugated anti-mouse and Alexa Fluor 594 conjugated anti-rabbit IgG secondary antibodies (Molecular Probes Invitrogen, Waltham, MA, USA, 1:100). IF micrographs were obtained at 400× magnification using a Zeiss LSM710 confocal microscope and Zen2009 software (Zeiss, Oberkochen, Germany).

The FACS procedure was performed as described [19] on NS preparations and trypsinized clones, and analysis was performed with a MoFlo Astrios cell sorter and Kaluza 2.1 software (both from Beckman Coulter, Miami, FL, USA). For FACS analysis the following antibodies were used: rabbit monoclonal anti-cytokeratin 7 (CK7, clone EPR1619Y, Abcam, Cambridge, UK, 1:20), mouse monoclonal APCH7-conjugated anti-CD10 (clone HI10a, Becton Dickinson, San Jose, CA, USA, 1:20), mouse monoclonal APC-conjugated anti-CD31 (clone WM59, BioLegend, San Diego, CA, USA, 1:20), mouse monoclonal FITC-conjugated anti pan CK (clone CK3-CH5, Miltenyi Biotech, Bergisch Gladbach, Germany, 1:10), mouse monoclonal APC-conjugated anti-CD133 (clone AC133, Miltenyi Biotech, Bergisch Gladbach, Germany, 1:10), mouse monoclonal FITC-conjugated anti CD24 (clone ML5, BioLegend, San Diego, CA, USA, 1:20). Alexa Fluor 488 conjugated anti-rabbit (Molecular Probes Invitrogen, Waltham, MA, USA, 1:100) was used as secondary antibody for cytokeratin 7.

### 2.4. FACS Sorting

The cell suspension obtained from PKH26 stained NS [14] was FACS sorted with a MoFlo Astrios cell sorter on the basis of PKH fluorescence intensity. We isolated the cellular population with the highest PKH fluorescence (PKH^high^) gated on the basis of the sphere forming efficiency (SFE) percentage, which is described to be around 1%. PKH^low/neg^ cells, with intermediate or without fluorescence, were gated as 80–90% of the total cell population [14]. Within the PKH^high^ cells, we also isolated the CD133+/CD24− cell subpopulation (RSC) by FACS sorting, described to be around 70% of PKH^high^ cells [14], and within the PKH^low/neg^ cells we isolated the CD31+ cells (gated as about 1%) and the CD31− cells (gated as about 90%). Single cell sorting of RSC and PKH^low/neg^ populations was performed on 96-well plates and the presence of a single cell per well was assessed under contrast phase microscope (Leica, Wetzlar, Germany). An average sorting rate of 500–1000 events per second at a sorting pressure of 25 psi with a 100 μm nozzle was maintained.

### 2.5. RSC Cultured on Decellularized Extracellular Matrix (ECM) Kidney Scaffolds and Three-Dimensional (3D) Staining

Frozen human renal tissues were cut into approximately 2-mm-thick slices maintaining all kidney regions. Slices were decellularized as described [16] and a portion of the scaffold was routinely tested for complete decellularization by Hematoxylin and Eosin (H&E) staining on multiple formalin fixed, paraffin embedded (FFPE) sections. 15,000 FACS sorted RSC were seeded on the decellularized renal scaffold, obtained from the same patient and cultured with basal medium (DMEM low glucose supplemented with 10% FBS, both from EuroClone, Milan, Italy) in 96-well poly-HEMA coated plates. Five different experiments, each representing one individual tissue patient were performed. The cells were allowed to attach to the ECM scaffold for 5 days while only adding the medium without changing it, then the medium was changed every 2 days. The cultures were stopped at 30 days, formalin fixed for at least 16 h and paraffin embedded for histological analysis or processed for 3D staining as follows:

A small portion of two of the different 30-day-cultured scaffolds was cut and fixed in formalin for 1 h. The two scaffold samples were incubated with Alexa Fluor 680–phalloidin (1:100 in PBS; Molecular Probes Invitrogen, Waltham, MA, USA) for 15 min and with 4’,6-diamidino-2-phenylindole (DAPI) (Sigma-Aldrich, St.Louis, MO, USA) for 10 min, then mounted between a glass coverslip and glass slides. Immunofluorescence micrographs were obtained at ×400 magnification using the Zeiss LSM710 confocal microscope and Zen2009 software (Zeiss, Oberkochen, Germany). Z-stack function was used to acquire sequential micrographs every 1.2 μm, covering the entire thickness of the chosen structures and then 3D reconstruction was assembled using the specific ImageJ software (NIH, Bethesda, MD; http://imagej.nih.gov/ij) 3D plugin.

### 2.6. Histologic Characterization

The decellularized scaffolds, on which RSC was cultured, were FFPE, sectioned (2-µm-thick) and H&E stained in order to assess the cellular repopulation and to evaluate the morphologic features. Images were acquired using the Hamamatsu NanoZoomer S60 scanner (Nikon, Campi Bisenzio, Italy). The S60 scanner has two six-filter wheels, one for excitation the other for emission filters, a three-cube turret and is equipped with a Plan Apochromat Lambda 20x NA 0.75 objective (Nikon, Campi Bisenzio, Italy), a Fluorescence Imaging Module L13820 equipped with a mercury lamp (Hamamatsu Photonics, Arese, Italy) and an ORCA-Flash 4.0 digital CMOS camera (Hamamatsu Photonics, Arese, Italy). IF staining of histologic sections was performed as described [16]^,^ using mouse monoclonal anti-aquaporin (AQP1, clone B-11, Santa Cruz, Dallas, TX, USA, 1:50), rabbit polyclonal anti-CD13 (Santa Cruz, Dallas, TX, USA, 1:200), mouse monoclonal anti-N-cadherin (NCAD, clone 32, Becton Dickinson, San Jose, CA, USA, 1:50) proximal tubular markers; rabbit monoclonal anti-cytokeratin 7 (CK7, clone EPR1619Y, Abcam; 1:200), mouse monoclonal anti-calbindin-D28k (CALB, clone CB-955, Sigma-Aldrich, St.Louis, MO, USA, 1:200), mouse monoclonal anti- E-cadherin (ECAD, clone 36, Becton Dickinson, San Jose, CA, USA, 1:50) distal tubular markers; mouse monoclonal anti-cytokeratin 8.18 (CK 8.18), epithelial marker (clone 5D3, 1:50); von Willebrand factor (vWf), endothelial marker (1:2000) primary antibodies and Alexa Fluor 680 conjugated goat anti-mouse and Alexa Fluor 594 conjugated goat anti-rabbit IgG secondary antibodies (1:100). DAPI dilactate 5.45 µM (Sigma-Aldrich, St.Louis, MO, USA) was added for nuclear counterstaining. The immunofluorescent images were acquired using the S60 scanner or the Zeiss LSM710 confocal microscope and Zen2009 software (Zeiss, Oberkochen, Germany). In cases of sequential IF staining, the sections were subjected to elution of the previous antibodies by incubation in a shaking water bath at 56 °C for 30 min in a 2% SDS, 114-mM 2-mercaptoethanol, 60-mM Tris-HCl pH 6.8 solution [20].

### 2.7. In Vitro Single Cell Differentiation

The single-sorted RSC were grown in different media. The epithelial medium consisted in DMEM-F12 (Lonza, Basel, Switzerland) supplemented with 10% FBS, ITS supplement (5-μg/mL insulin, 5-μg/mL Transferrin, 5-ng/mL sodium selenite), 36-ng/mL hydrocortisone, 40-pg/mL triiodothyronine (all from Sigma-Aldrich, St.Louis, MO, USA), 20-ng/mL EGF (cell-signaling) and 50-ng/mL HGF (cell-signaling). The endothelial medium was composed of endothelial cell basal medium (EBM™, Lonza, Basel, Switzerland) supplemented with EGM™SingleQuots™ (Lonza, Basel, Switzerland), 10% fetal bovine serum. FACS analysis with the appropriate antibodies was performed on the trypsinized cells of obtained clones.

### 2.8. 3D Culture on Matrigel and Staining

The 96-well plates were precoated with 75 µl of 10-mg/mL growth factor reduced, phenol red free Matrigel^®^ matrix (Corning, Midland County, MI, USA). Then, 10,000 cells each of the following RSC, CD31−/PKH^low/neg^, CD31+/PKH^low/neg^ cell subpopulations were FACS sorted after dissociation of NS and seeded on Matrigel in the endothelial medium described above to perform morphogenesis assay as described [21]. Contrast-phase images were obtained at 6 h and at different time points starting at 24 h to follow the growth. Matrigel plugs were subjected to 3D IF staining with a protocol modified from Lee et al. [22]. Plugs were fixed with 4% paraformaldehyde (Thermo Fisher scientific, Waltham, MA, USA) at room temperature (RT) for 10 min and treated with PBS glycine (100 mM) (EuroClone, Milan, Italy) for other 10 min and then incubated with 0.5 mg/mL of proteinase K (Sigma-Aldrich, St.Louis, MO, USA) for 5 min at RT. A blocking solution composed by IF buffer (0.5% Triton, 0.1% BSA, 0.05% Tween20 in PBS) with 10% normal goat serum (EuroClone, Milan, Italy) was added for 1 h and half at RT with shaking. Incubation with rabbit monoclonal anti CK8.18 (clone EP17/EP30, DAKO, Copenhagen, Denmark, 1:25), mouse monoclonal CD31 (clone JC70A, DAKO, Copenhagen, Denmark, 1:25), mouse monoclonal E-cadherin (clone 36, Becton Dickinson, San Jose, CA, USA, 1:50) and rabbit polyclonal vWf primary antibodies diluted in IF buffer was performed at RT for 2 h and then with Alexa Fluor 488 conjugated goat anti-mouse and Alexa Fluor 594 conjugated goat anti-rabbit IgG secondary antibodies (1:100). IF micrographs were obtained at 400x magnification using a Zeiss LSM710 confocal microscope and Zen2009 software (Zeiss, (Oberkochen, Germany).

### 2.9. Statistical Analysis.

For FACS analysis of CD31 and CK7/CD10 expression, the chi-squared test was used to compare the overall proportion of positive cells among the three subpopulations PKH^high^, PKH^low^ and PKH^neg^. Pairwise comparisons were performed again with the chi-squared test with Holm correction for multiplicity. These tests take into account the differences in the numbers of the analyzed events between the three cell subpopulations examined. The chi-squared test was again used for the FACS analysis of CD31 and pan cytokeratin expression on the cells within the clones cultured in epithelial or endothelial medium to assess the statistical differences and *p* < 0.01 was considered significant.

## 3. Results

### 3.1. Human RSC Cultured on Human Decellularized Scaffolds

**Figure 1:**
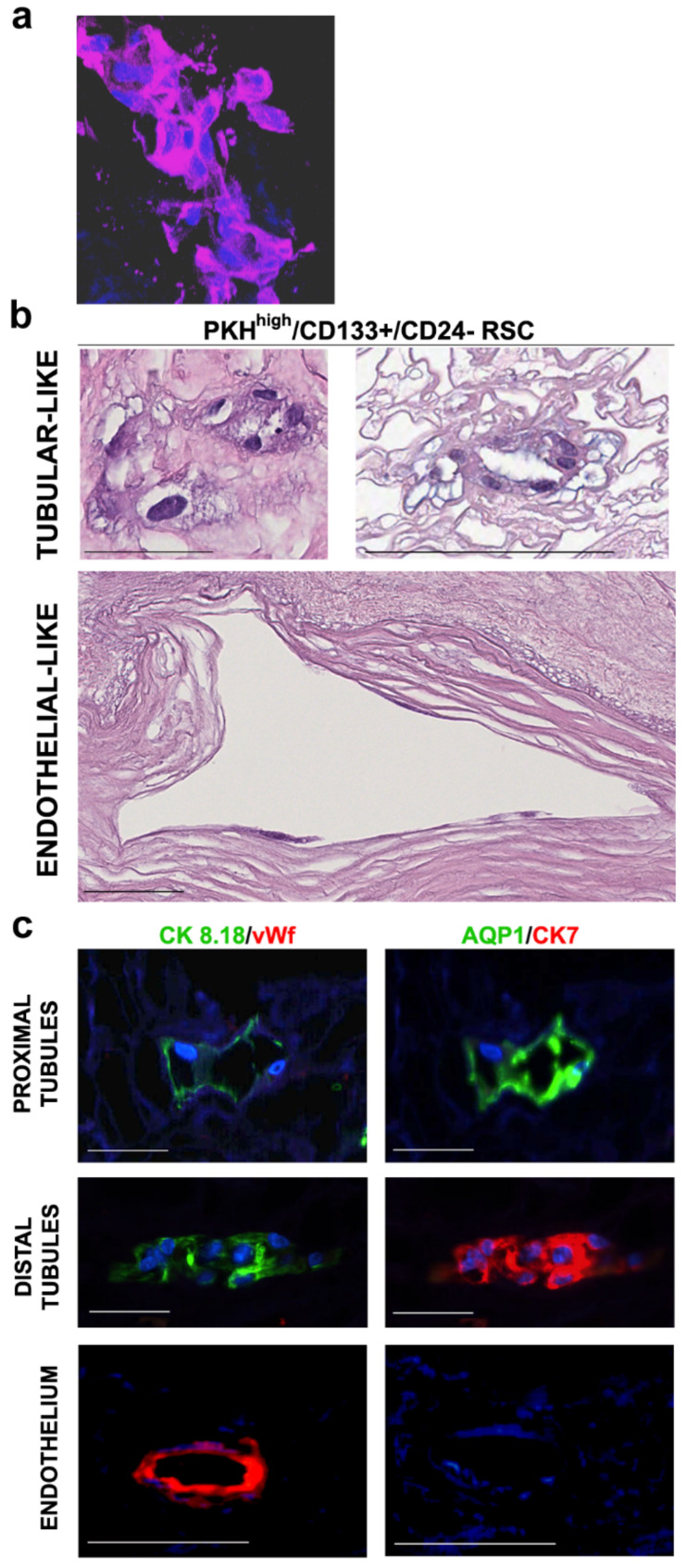
Histological characterization of the decellularized scaffolds repopulated with adult renal stem-like PKH^high^/CD133+/CD24− cells (RSC). (**a**) Representative Z-stack 3D reconstruction of images after DAPI (blue) and phalloidin (purple) immunofluorescence staining of the scaffolds at 30 days after RSC seeding. Total thicknesses of the shown structure was 31.2-µm original magnification, ×400; (**b**–**c**) five independent experiments of human kidney scaffold repopulation for 30 days with RSC in presence of basal medium were performed; (**b**) representative Hematoxylin and Eosin (H&E) staining of formalin fixed, paraffin embedded (FFPE) repopulated scaffold sections. Scale bars, 100 µm; (**c**) representative sequential IF analysis of the FFPE-repopulated scaffolds with the antibodies against the indicated markers that recognize specific tubular or vascular phenotypes. The different antibodies combinations identify proximal tubules (top), distal tubules (middle) and endothelium (bottom). Scale bars, 50 µm. CK—Cytokeratin; AQP—Aquaporin; vWf—Von Willebrand Factor; blue—DAPI.

RSC (PKH^high^/CD133+/CD24− cells) were obtained by FACS sorting from NS cultured for 10 days. PKH^high^ cells represent the PKH26 most fluorescent cells, gated as about 0.8–1% of the total NS population. This percentage of PKH^high^ cells corresponds to the usual sphere forming efficiency (SFE) observed [14]. The RSC were gated as about 70% of PKH^high^ cells on the basis of CD133+/CD24− phenotype [14] and represented those with higher stem-like capacity PKH^high^/CD133+ cells, while the PKH^high^/CD133− cells do not have self-renewal capacity [14]. We seeded 15,000 sorted cells of RSC (the maximum number of yielded cells) on decellularized scaffolds placed in poly-HEMA coated 96-well plates [16]. After 5 days the cells, which could not attach to the plastic because of poly-HEMA, were into the scaffold since no cells were observed in the area of well not covered by the scaffold. The limited number of cells did not repopulate completely the scaffold, however, our aim was to investigate the differentiation abilities of RSC and their capability to settle in specific nephron portions and not to entirely repopulate the scaffolds. With this purpose, the small number of cells on the scaffold had the advantage to mimic a situation close to a clonal-like condition in which theoretically single cells could attach, proliferate and differentiate to generate a specific structure. The 3D staining of small scaffold portions, after 30 days of culture with RSC in presence of a basal medium without any other additional growth factor, showed the presence of cells demonstrating the nontoxicity of the decellularized matrix and the capability of the RSC to proliferate and differentiate into the scaffolds (Figure 1a). In addition, the RSC showed the capacity to localize at different specific portions of the scaffold, as evidenced by H&E staining (Figure 1b). H&E showed the presence of simple cuboidal epithelial-like cells, typical of the renal tubular portion (Figure 1b, top panels) as well as flat cells, similar to simple squamous endothelial-like cells, located on the big vessel basement membrane (Figure 1b, bottom panels). No cells were visible after 30 days of culture in the control scaffold in which we did not plate any cells (Supplementary Figure S1a).

Using the sequential IF staining [21], which permitted us to evaluate several markers on the same repopulated structure, we documented that the different morphology acquired in the scaffold by RSC corresponded to different renal cell phenotypes. This capacity, already described for the whole cells present in NS [16], is now demonstrated to be associated with the stem-like cells. The epithelial (CK8.18), proximal (AQP1, CD13 and N-cadherin) and distal (CK7, calbindin D-28k and E-cadherin) tubular and endothelial (vWf) markers were evaluated. RSC, after 30 days of culture on decellularized scaffolds, mostly generated cells showing epithelial proximal or distal tubular- or endothelial-like phenotype localized on the tubular or vascular spaces of the scaffold, respectively (Figure 1c). In fact, the cells in tubular structures expressed the epithelial CK8.18 and proximal and distal tubular markers in a mutually exclusive way indicating a specific lineage differentiation (Figure 1c, upper and middle panels; Supplementary Figure S3). Endothelial vWf was not detectable in the structures with cells expressing tubular markers (Figure 1c, upper and middle panels). Instead, other structures had cells expressing endothelial vWf, lacking the expression of CK8.18 epithelial and AQP1 and CK7 tubular markers (Figure 1c, lower panels). Of the structures formed by RSC, 75% presented epithelial-like phenotype and 18% presented endothelial-like phenotype. Of note, few structures (8%) repopulated with RSC presented cells that co-expressed markers of different lineages, suggesting a still immature cell phenotype or a transitional state. In fact, some of them co-expressed epithelial CK8.18, tubular AQP1/CK7 and endothelial vWf markers (Supplementary Figure S1b). These data show that RSC organized in structures containing cells expressing epithelial or endothelial markers, supporting the multipotency of these progenitors isolated from human kidneys.

PKH^low/neg^ progenitors were cultured on decellularized scaffolds as controls. H&E staining showed only the presence of simple cuboidal epithelial-like cells, typical of the renal tubular portion and not of endothelial-like cells (Supplementary Figure S2a). Sequential immunofluorescence staining confirmed the epithelial tubular-like phenotype. In fact, PKH^low/neg^ cells, after 30 days of culture on decellularized scaffolds, only generated cells showing epithelial proximal or distal tubular-like phenotype. No vWf+ cells within the structures was observed. In tubular structures were present cells expressing the epithelial CK8.18 and tubular markers, proximal AQP1 or distal CK7, in a mutually exclusive way indicating a specific lineage differentiation (Supplementary Figure S2b) or together indicating a still immature phenotype (Supplementary Figure S2c).

### 3.2. Cell Commitment Toward Epithelium and Endothelium

**Figure 2:**
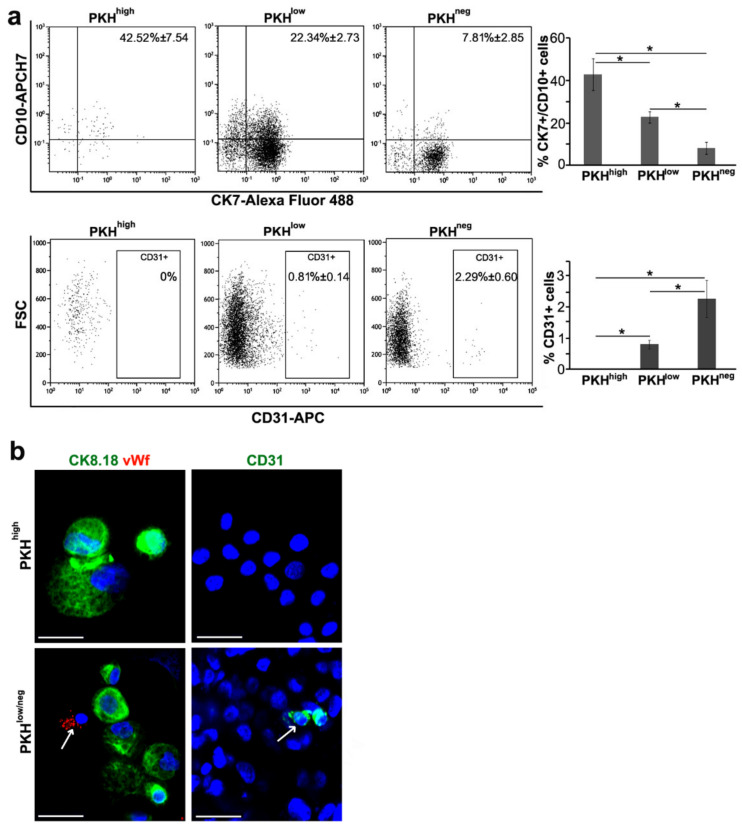
Immunophenotypical characterization of nephrosphere cell subpopulations. (**a**) FACS analysis of nephrospheres (NS) cell subpopulations PKH^high^, PKH^low^, PKH^neg^ gated on PKH26 fluorescence. PKH^high^ cells were the brightest PKH-positive cells and gated as 0.8–1% of the total population. PKH^low^ cells, with intermediate fluorescence, were gated as 15–20% of the total cell population and the PKH^neg^ cells, without fluorescence, as 60–70% of the total cell population. The antibodies against the indicated markers were used. For each subpopulation, the overall weighted-mean percentages ± SD referred to three (top) and four (bottom) independent experiments, are reported in the dot plots. CK—cytokeratin; FSC—forward scatter. Chi-squared test, *p < 0.001; (**b**) immunofluorescence (IF) analysis of PKH^high^, PKH^low/neg^ cells sorted from dissociated NS and then cytospinned. The indicated markers were evaluated. Blue—DAPI. Results are representative of at least three independent experiments. Original magnification 400×. Scale bars, 50 µm. Arrows: vWf+ (left panel) and CD31+ (right panel) cells.

The PKH^high^ cells obtained dissociating NS showed the co-expression of proximal CD10 and distal CK7 tubular markers (42.53% ± 7.54 weighted mean ± standard deviation), which dramatically decreased in PKH^low^ (22.34% ± 2.73) and PKH^neg^ (7.81% ± 2.85) cells (Figure 2a, upper panels), suggesting the intrinsic plasticity of these progenitors. The co-expression of CD10 and CK7 markers was also observed in RSC, gated as about 70% of PKH^high^ cells, before seeding on decellularized scaffold (data not shown). More surprisingly, although the PKH^high^ cells did not express the endothelial CD31 marker (0%), their PKH^low^ and PKH^neg^ cell progeny inside the NS expressed CD31 (0.81% ± 0.14% and 2.29% ± 0.60, respectively), suggesting that CD31 expression increased within the spheres in relation to proliferation assessed by PKH26 dye fluorescence decrement (Figure 2a, lower panels). Even on cytospinned PKH^high^ cells, the total negativity of vWf and CD31 endothelial markers and the positivity of epithelial marker CK8.18 was documented (Figure 2b, upper panels). On the contrary, the presence of few cells with an endothelial phenotype (vWF+ and CD31+) was confirmed in the cytospinned PKH^low/neg^ cells (Figure 2b, white arrow in lower panels). About 20% of the CD31+ cells in the PKH^low^ and PKH^neg^ cells showed a co-expression with cytokeratin (data not shown), indicating a transitional state of differentiation inside the NS that we already noted in repopulated scaffold structures (Supplementary Figure S1b). Therefore, these data suggest the existence of a differentiation gradient within the NS starting from immature PKH^high^ cells toward different lineage commitment evidenced in PKH^low^ and PKH^neg^ progeny.

**Figure 3:**
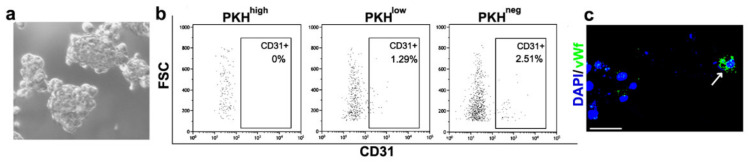
Characterization of secondary NS generated from FACS-sorted CD31- RSC. (**a**) Contrast-phase image of secondary NS generated from CD31- RSC FACS sorted from primary NS; (**b**) FACS analysis of CD31 expression in PKH^high^, PKH^low^, PKH^neg^ cell subpopulations of secondary NS cells gated on PKH26 fluorescence; (**c**) IF analysis of cytospinned cells obtained from secondary NS dissociation. Antibody against vWf was used. Blue—DAPI. Original magnification 400×. Scale bar, 50 µm. Arrow: vWf+ cells.

To demonstrate that the RSC, about 70% of PKH^high^ cells, have the capacity to generate the CD31+ cells, we sorted CD31- RSC from NS and plated them for ten days to form new secondary NS (Figure 3a). FACS analysis of cells from these secondary NS revealed again that the PKH^high^ cells retained the CD31 phenotype, while only some of PKH^low^ and PKH^neg^ cell progeny expressed CD31 (Figure 3b), likely due to the presence of a lineage commitment within NS. Even in secondary NS, as in primary NS (Figure 2a), there was a gradient of enrichment in CD31+ cells along proliferation/differentiation from the quiescent PKH^high^ cells to the proliferating PKH^low^ and PKH^neg^ cells. Furthermore, the expression of the endothelial marker vWf was detected by IF in some cells of secondary NS generated from the RSC negative for endothelial markers (Figure 3c). These data support the observation that RSC were able to give rise to endothelial cells, suggesting an intrinsic multipotency capacity.

### 3.3. Morphogenesis 3D Assay

**Figure 4:**
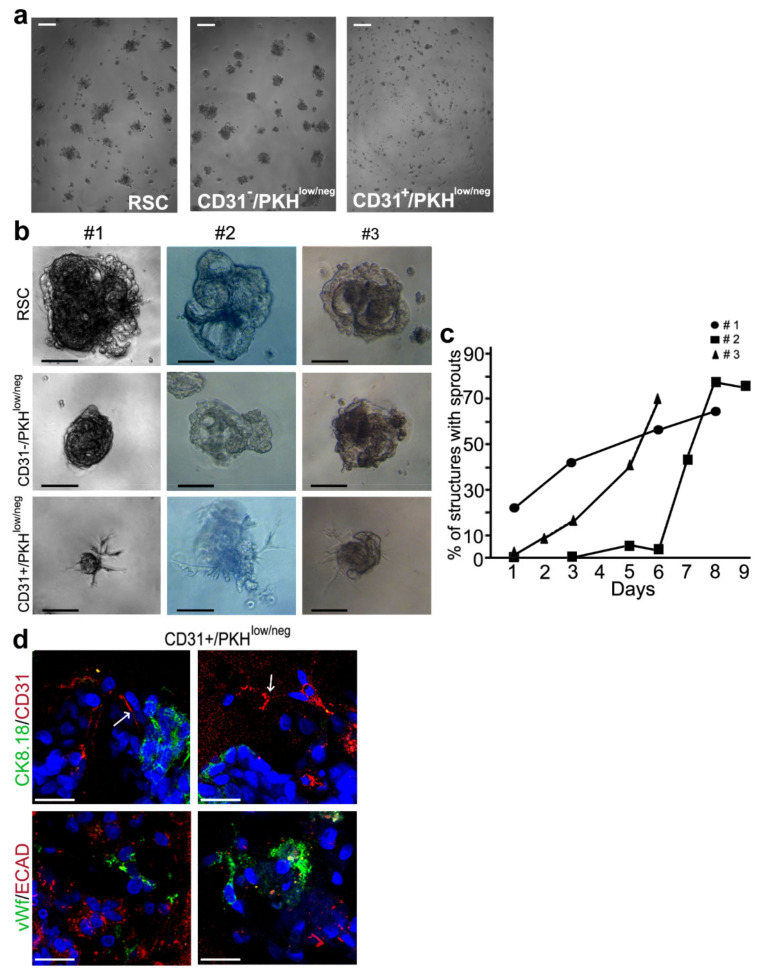
Morphogenesis 3D assay. (**a**) Contrast-phase images of RSC (left), CD31−/PKH^low/neg^ (middle) and CD31+/PKH^low/neg^ (right) at 6 h of culture. Scale bars, 100 µm; (**b**) contrast-phase images of structures obtained in Matrigel at 6–7 days of culture from the 3 different samples #1, #2, #3 of RSC (top), CD31−/PKH^low/neg^ (middle), CD31+/PKH^low/neg^ (bottom). Scale bars, 100 µm; (**c**) percentage of structures with sprouts in the three different samples of CD31+/PKH^low/neg^ at different time points; (**d**) 3D IF staining of the structures obtained from CD31+/PKH^low/neg^ cells with the antibodies against the indicated markers. Two different fields for each staining are shown. CK: cytokeratin; ECAD: E-cadherin; vWf: von Willebrand Factor; Blue—DAPI. Original magnification 400×. Scale bars, 50 µm. Arrows: CD31+ cells in the sprout-like extensions.

The behavior of the endothelial committed cells within the NS was analyzed by a morphogenesis 3D assay in Matrigel using three different cell subpopulations sorted from NS. We analyzed the CD31− RSC, the CD31−/PKH^low/neg^ progenitors and the CD31+/PKH^low/neg^ progenitors committed to an endothelial lineage. At 6 h of culture, all our cell subpopulations started organizing as spheroids (Figure 4a). Extending the time of culture, only the endothelial-committed CD31+/PKH^low/neg^ population generated sprouts similar to capillary-like structures [23] (Figure 4b) at different time points and in the following days these sprouts increased over time (Figure 4c). RSC generated bigger and more organized spheroids than CD31−/PKH^low/neg^ cells and both without any sprouts (Figure 4b, upper and middle panels). In addition, 3D IF staining evidenced the presence of CD31+ and vWf+ cells in structures generated by CD31+/PKH^low/neg^ progenitors. We did not observe the colocalization of CD31 and vWf endothelial markers with CK8.18 and E-cadherin epithelial markers (Figure 4d). CD31 was localized in the sprout-like extensions, as indicated by the arrows (Figure 4d, upper panels). This morphogenesis assay provided further observations indicating that in NS the CD31− RSC can generate endothelial committed CD31+/PKH^low/neg^ progenitor cells, which are able to differentiate into an endothelial-like phenotype.

### 3.4. Single Cell Differentiation

**Figure 5:**
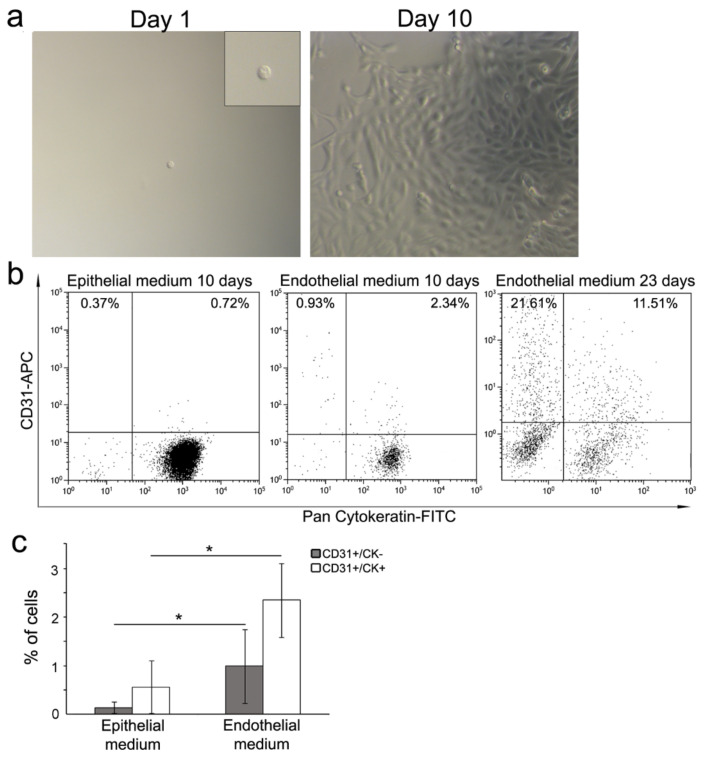
Single cell differentiation. (**a**) Contrast-phase images of a representative single-sorted RSC (left panel) and the representative clone generated at day 10. Original magnification: 100×. Insert: 2X digital zoom; (**b**) FACS analysis of two representative clones obtained from single RSC at ten days of culture in epithelial (left panel) and endothelial (middle panel) media. FACS analysis at 23 days of culture of one representative clone in endothelial medium (right panel). The CD31 and cytokeratin markers were evaluated; (**c**) graphic representation of CD31+/CK- and CD31+/CK+ cell percentage within the clones obtained after 10 days of culture in specific media. Weighted mean ± SD is referred to four independent experiments of 10 clones grown in epithelial medium and 6 in endothelial medium. Chi-squared test, *p < 0.01.

To confirm the multipotency ability of CD31- RSC, their capacity to differentiate into both tubular epithelial and endothelial lineages was assessed at single cell level in 2D culture. Starting from NS, obtained from three different patients, one single RSC was sorted into wells containing specific epithelial (48 wells) or endothelial (48 wells) media. One 96-well plate for each patient sample was used, and the single cell presence in the wells was assessed under contrast phase microscope (Figure 5a, left panel). We were able to obtain cell clones (Figure 5a, right panel) from 34% (range 18.7–62.5%) of single RSC in epithelial medium and from 22% (range 12.5–37.5%) of single RSC in endothelial medium. The expression of cytokeratin epithelial marker and CD31 endothelial marker was evaluated by FACS at 10 days of confluent clone culture. With both media, most of the cells (>90%) evaluated in 16 of the obtained clones expressed cytokeratin, indicating the commitment toward epithelial lineage (Figure 5b). In all 16 clones tested, a few cells expressed CD31 alone (range 0.05–2.83%) or together with cytokeratin (range 0.12–4.72%), indicating a differentiation toward endothelial lineage or a transitional status, respectively (Figure 5b, left and middle panels). However, the mean percentage of CD31+/CK- endothelial cells as well CD31+/CK+ cells was significantly higher in the clones obtained in endothelial medium (0.99% ± 0.76% and 2.35% ± 0.76, respectively, weighted mean ± SD) compared to those in epithelial medium (0.14 ± 0.11 and 0.56 ± 0.54, respectively) (Figure 5c). Extending the culture time to 23 days for two clones grown in endothelial medium, the percentage of CD31+ cells was much higher than 10 days (Figure 5a, right panel). With this experiment we demonstrate that from one single CD31− RSC it was possible to obtain clones that contain both epithelial like (CK+) and endothelial like (CD31+) cells, as evidence of the multipotency of the single RSC. This capacity is unique to RSC, since single PKH^low/neg^ progenitor cells can generate clones containing only epithelial like (CK+) cells (Supplementary Figure S2d).

## 4. Discussion

The aim of this study was to investigate the in vitro multipotency of human adult renal stem-like PKH^high^/CD133+/CD24− cells (RSC) using different in vitro tests. Our data evidenced that RSC subpopulation, able to generate NS, had the capacity to attach to tubular and vascular segments of renal scaffolds differentiating in epithelial- and endothelial-like lineages, respectively. RSC were cultured on renal scaffold with a medium without any growth factor or external stimuli, so RSC may have proliferated and differentiated responding to the growth factors preserved in the ECM [23,24] as well as to the basement membrane composition [25].

RSC did not present any ability to repopulate the glomerular endothelium and epithelium. In fact, visceral epithelium and a differentiation towards podocytes were not present, although we previously demonstrated that RSC were able to differentiate in vitro into podocytes upon appropriate stimuli [14]. In our model, RSC are seeded on the scaffold in a static system and do not integrate into the glomerulus probably due to the physical obstacle represented by the capillary matrix [26]. Moreover, it is described [27] that glomerular endothelium appears important for the induction of the final maturation of podocytes. A few structures presented cells co-expressing markers, which are specific for these different lineages of differentiation, as a possible evidence of a transient and immature phenotype.

In addition, RSC not expressing CD31 and vWf endothelial markers were able to generate NS, which contained some CD31+ filial cells. These CD31+ cells, sorted from NS, generated in Matrigel 3D spheroids that branched in capillary-like sprouts exhibiting an endothelial-like behavior. In fact, spheroid sprouting is typical of endothelial cells and it is considered a reliable 3D angiogenesis assay [28]. The fact that only spheroids obtained from CD31+ PKH^low/neg^ cells could generate sprouts in a 3D assay can be an indication of their endothelial commitment. PKH^high^ cells were also enriched in cells co-expressing proximal and distal tubular markers. This co-expression decreased in the PKH^low/neg^ cells, the progeny of PKH^high^ cells, while CD31+ cells increased. These data may indicate a differentiation gradient within NS, in which PKH^low/neg^ progeny represents the subpopulation committed to proximal and distal tubular and endothelial phenotype. The presence of a differentiation gradient inside the spheres was also described in mammospheres [29]. Moreover, the capacity of a single RSC to commit into both epithelial and endothelial lineage was demonstrated by an in vitro single cell differentiation assay. This commitment of single RSC into both epithelial and endothelial lineages was surprising. In fact, we were dealing with a single adult epithelial stem-like cell that was not expected to differentiate into endothelial lineage. Multipotent capacity was demonstrated to be unique of RSC, since PKH^low/neg^ progenitors are neither able to generate endothelial-like structures on the scaffolds nor to exhibit multipotency at single cell level.

The repeated appearance of this result indicates that the endothelial commitment and differentiation of RSC, which lack not only endothelial, but also mesenchymal and hematopoietic markers (data not shown), can be possible. There is some support for this possibility in literature. Little and McMahon [30], reviewing the development of mammalian kidney, assessed that vascular progenitors exist within the metanephric mesenchyme and may actively populate newly forming vasculature through a vasculogenic process. In addition, 3D organoids obtained from iPSC reprogrammed into intermediate mesoderm contain both epithelial and vascular compartments [31,32]. Therefore, we can speculate that also our RSC, though they are adult stem cells, can somehow acquire multipotency and differentiate into epithelial and endothelial lineages, even though there is no current evidence of such a behavior in vivo in lineage-tracing mouse models [4,8]. A possible explanation of this observed intriguing phenomenon of multipotency can be found in the study published by Gonçalves and colleagues [33]. The authors evidenced that angiomyolipoma cells derive from a multipotent cancer stem cell that is generated by a renal epithelial cell. Becherucci and Romagnani commented on this study, suggesting the possibility that in the renal epithelium there may be a differentiation capacity that goes behind the epithelial phenotype [34]. In fact, it was proposed that in different conditions, specific stem cells can be exposed to factors that are different from those of their specific tissue niches [35]. In the same way, renal epithelial stem cells could exhibit lineage restricted capacity when evaluated with in vivo models of lineage tracing under their steady-state conditions, but in some different conditions, such as in vitro culture, they could show cellular plasticity. We can thus hypothesize that multipotency capacity is something intrinsic, but dormant in resident stem cells in the healthy human kidney, while the NS microenvironment can establish a new niche exposing the RSC to new and different factors able to rouse multipotency capacities.

Based on these observations, our model can be promising because it allows us to isolate a plastic population of human renal stem-like cells which is able to grow as NS and to recapitulate the development of different renal lineages. In this scenario, the RSC can be a good tool for the creation of renal organoids and the ability of RSC to differentiate into endothelium and epithelium may eventually have translational relevance for studying the parenchymal and endothelial cell–cell interactions and developing new therapeutic strategies.

## Supplementary Materials

The following are available online at https://www.mdpi.com/2073-4409/9/8/1805/s1, Figure S1: Histological characterization of decellularized scaffolds repopulated with RSC; Figure S2: Differentiation abilities of PKH^low/neg^ progenitor cells; Figure S3: Histological characterization of decellularized scaffolds repopulated with RSC.

## References

[1] Prescott L.F. (1966). The normal urinary excretion rates of renal tubular cells, leucocytes and red blood cells. Clin. Sci..

[2] Lameire N.H., Bagga A., Cruz D., de Maesneer J., Endre Z., Kellum J.A., Liu K.D., Mehta R.L., Pannu N., van Biesen W. (2013). Acute kidney injury: An increasing global concern. Lancet.

[3] Humphreys B.D., Valerius M.T., Kobayashi A., Mugford J.W., Soeung S., Duffield J.S., McMahon A.P., Bonventre J.V. (2008). Intrinsic epithelial cells repair the kidney after injury. Cell Stem Cell.

[4] Rinkevich Y., Montoro D.T., Contreras-Trujillo H., Harari-Steinberg O., Newman A.M., Tsai J.M., Lim X., Van-Amerongen R., Bowman A., Januszyk M. (2014). In vivo clonal analysis reveals lineage-restricted progenitor characteristics in mammalian kidney development, maintenance, and regeneration. Cell Rep..

[5] Johnson H.A., Vera-Roman J.M. (1996). Compensatory renal enlargement. Hypertrophy versus hyperplasia. Am. J. Pathol..

[6] Lombardi D., Becherucci F., Romagnani P. (2016). How much can the tubule regenerate and who does it? An open question. Nephrol. Dial. Transplant..

[7] Pleniceanu O., Omer D., Harari-Steinberg O., Dekel B. (2018). Renal lineage cells as a source for renal regeneration. Pediatr. Res..

[8] Lazzeri E., Angelotti M.L., Peired A., Conte C., Marschner J.A., Maggi L., Mazzinghi B., Lombardi D., Melica M.A., Nardi S. (2018). Endocycle-related tubular cell hypertrophy and progenitor proliferation recover renal function after acute kidney injury. Nat. Commun..

[9] Bussolati B., Bruno S., Grange C., Buttiglieri S., Deregibus M.C., Cantino D., Camussi G. (2005). Isolation of renal progenitor cells from adult human kidney. Am. J. Pathol..

[10] Bussolati B., Moggio A., Collino F., Aghemo G., D’Armento G., Grange C., Camussi G. (2012). Hypoxia modulates the undifferentiated phenotype of human renal inner medullary CD133+ progenitors through Oct4/miR-145 balance. Am. J. Physiol. Renal. Physiol..

[11] Sagrinati C., Netti G.S., Mazzinghi B., Lazzeri E., Liotta F., Frosoli F., Ronconi E., Meini C., Gacci M., Squecco R. (2006). Isolation and characterization of multipotent progenitor cells from the Bowman’s capsule of adult human kidneys. J. Am. Soc. Nephrol..

[12] Angelotti M.L., Ronconi E., Ballerini L., Peired A., Mazzinghi B., Sagrinati C., Parente E., Gacci M., Carini M., Rotondi M. (2012). Characterization of renal progenitors committed toward tubular lineage and their regenerative potential in renal tubular injury. Stem Cells.

[13] Bussolati B., Camussi G. (2015). Therapeutic use of human renal progenitor cells for kidney regeneration. Nat. Rev. Nephrol..

[14] Bombelli S., Zipeto M.A., Torsello B., Bovo G., di Stefano V., Bugarin C., Zordan P., Viganò P., Cattoretti G., Strada G. (2013). PKH^high^ cells within clonal human nephrospheres provide a purified adult renal stem cell population. Stem Cell Res..

[15] Steinberg O.H., Omer D., Gnatek Y., Pleniceanu O., Goldberg S., Cohen-Zontag O., Pri-Chen S., Kanter I., Ben-Haim N., Becker E. (2020). Ex Vivo Expanded 3D Human Kidney Spheres Engraft Long Term and Repair Chronic Renal Injury in Mice. Cell. Rep..

[16] Bombelli S., Meregalli C., Scalia C., Bovo G., Torsello B., de Marco S., Cadamuro M., Viganò P., Strada G., Cattoretti G. (2018). Nephrosphere-derived cells are induced to multilineage differentiation when cultured on human decellularized kidney scaffolds. Am. J. Pathol..

[17] Bianchi C., Bombelli S., Raimondo F., Torsello B., Angeloni V., Ferrero S., di Stefano V., Chinello C., Cifola I., Invernizzi L. (2010). Primary cell cultures from human renal cortex and renal-cell carcinoma evidence a differential expression of two spliced isoforms of Annexin A3. Am. J. Pathol..

[18] Torsello B., Bianchi C., Meregalli C., di Stefano V., Invernizzi L., de Marco S., Bovo G., Brivio R., Strada G., Bombelli S. (2016). Arg tyrosine kinase modulates TGF-β1 production in human renal tubular cells under high-glucose conditions. J. Cell Sci..

[19] di Stefano V., Torsello B., Bianchi C., Cifola I., Mangano E., Bovo G., Cassina V., de Marco S., Corti R., Meregalli C. (2016). Major Action of Endogenous Lysyl Oxidase in Clear Cell Renal Cell Carcinoma Progression and Collagen Stiffness Revealed by Primary Cell Cultures. Am. J. Pathol..

[20] Bolognesi M.M., Manzoni M., Scalia C.R., Zannella S., Bosisio F.M., Farretta M., Cattoretti G. (2017). Multiplex Staining by Sequential Immunostaining and Antibody Removal on Routine Tissue Sections. J. Histochem. Cytochem..

[21] Bruno S., Bassani B., D’Urso D.G., Pitaku I., Cassinotti E., Pelosi G., Boni L., Dominioni L., Noonan D.M., Mortara L. (2018). Angiogenin and the MMP9-TIMP2 axis are up-regulated in proangiogenic, decidual NK-like cells from patients with colorectal cancer. FASEB J..

[22] Lee G.Y., Kenny P.A., Lee E.H., Bissell M.J. (2007). Three-dimensional culture models of normal and malignant breast epithelial cells. Nat. Methods.

[23] Peloso A., Ferrario J., Maiga B., Benzoni I., Bianco C., Citro A., Currao M., Malara A., Gaspari A., Balduini A. (2015). Creation and implantation of acellular rat renal ECM-based scaffolds. Organogenesis.

[24] Caralt M., Uzarski J.S., Iacob S., Obergfell K.P., Berg N., Bijonowski B.M., Kiefer K.M., Ward H.H., Wandinger-Ness A., Miller W.M. (2015). Optimization and critical evaluation of decellularization strategies to develop renal extracellular matrix scaffolds as biological templates for organ engineering and transplantation. Am. J. Transplant..

[25] Sciancalepore A.G., Portone A., Moffa M., Persano L., de Luca M., Paiano A., Sallustio F., Schena F.P., Bucci C., Pisignano D. (2016). Micropatterning control of tubular commitment in human adult renal stem cells. Biomaterials.

[26] Remuzzi A., Figliuzzi M., Bonandrini B., Silvani S., Azzollini N., Nossa R., Benigni A., Remuzzi G. (2017). Experimental Evaluation of Kidney Regeneration by Organ Scaffold Recellularization. Sci. Rep..

[27] Pavenstädt H., Kriz W., Kretzler M. (2003). Cell biology of the glomerular podocyte. Physiol. Rev..

[28] Heiss M., Hellström M., Kalén M., May T., Weber H., Hecker M., Augustin H.G., Korff T. (2015). Endothelial cell spheroids as a versatile tool to study angiogenesis in vitro. FASEB J..

[29] Pece S., Tosoni D., Confalonieri S., Mazzarol G., Vecchi M., Ronzoni S., Bernard L., Viale G., Pelicci P.G., di Fiore P.P. (2010). Biological and molecular heterogeneity of breast cancers correlates with their cancer stem cell content. Cell.

[30] Little M.H., McMahon A.P. (2012). Mammalian kidney development: Principles, progress, and projections. Cold Spring Harb. Perspect. Biol..

[31] Takasato M., Er P.X., Chiu H.S., Maier B., Baillie G.J., Ferguson C., Parton R.G., Wolvetang E.J., Roost M.S., Chuva-de-Sousa-Lopes S.M. (2015). Kidney organoids from human iPS cells contain multiple lineages and model human nephrogenesis. Nature.

[32] Homan K.A., Gupta N., Kroll K.Y., Kolesky D.B., Skylar-Scott M., Miyoshi T., Mau D., Valerius M.T., Ferrante T., Bonventre J.V. (2019). Flow-enhanced vascularization and maturation of kidney organoids in vitro. Nat. Methods.

[33] Gonçalves A.F. (2017). Evidence of renal angiomyolipoma neoplastic stem cells arising from renal epithelial cells. Nat. Commun..

[34] Becherucci F., Romagnani P. (2018). Angiomyolipoma: A link between stemness and tumorigenesis in the kidney. Nat. Rev. Nephrol..

[35] Batlle E., Clevers H. (2017). Cancer stem cells revisited. Nat. Med..

