# Supplementary material for: PKHhigh/CD133+/CD24− Renal Stem-Like Cells Isolated from Human Nephrospheres Exhibit In Vitro Multipotency
Source: Cells. 2020 Jul 29;9(8):1805. doi: 10.3390/cells9081805 (PMC7465083; doi:10.3390/cells9081805)
Supplement: Supplementary file 1 [file cells-09-01805-s001.pdf]

SUPPLEMENTARY FILE MS 812089 Bombelli S. et al.

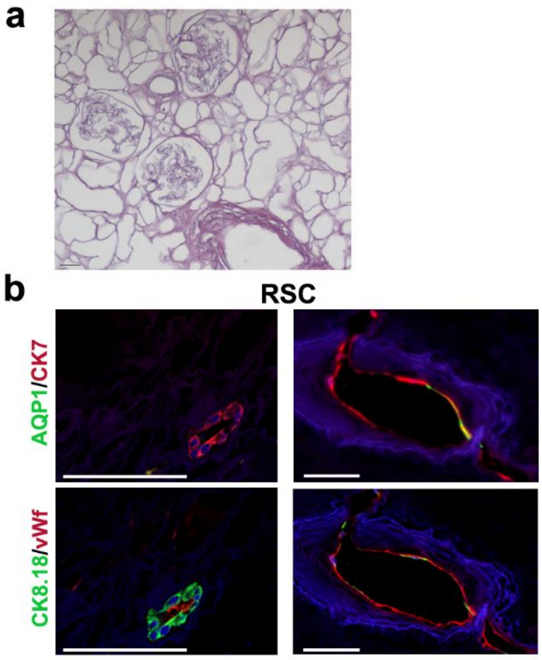

Supplementary Figure S1: Histological characterization of decellularized scaffolds repopulated with RSC. (**a**) Representative H&E staining of FFPE scaffold sections after 30 days without seeding any cells. Scale bars, 100 µm. (**b**) Representative IF analysis of FFPE scaffolds repopulated with RSC. The antibodies against the indicated markers recognise cells that co-express specific tubular epithelial and endothelial markers. Sequential immunostaining has been performed. Scale bars, 100 µm. CK: Cytokeratin; AQP: Aquaporin; vWf: von Willebrand Factor; Blue:DAPI.

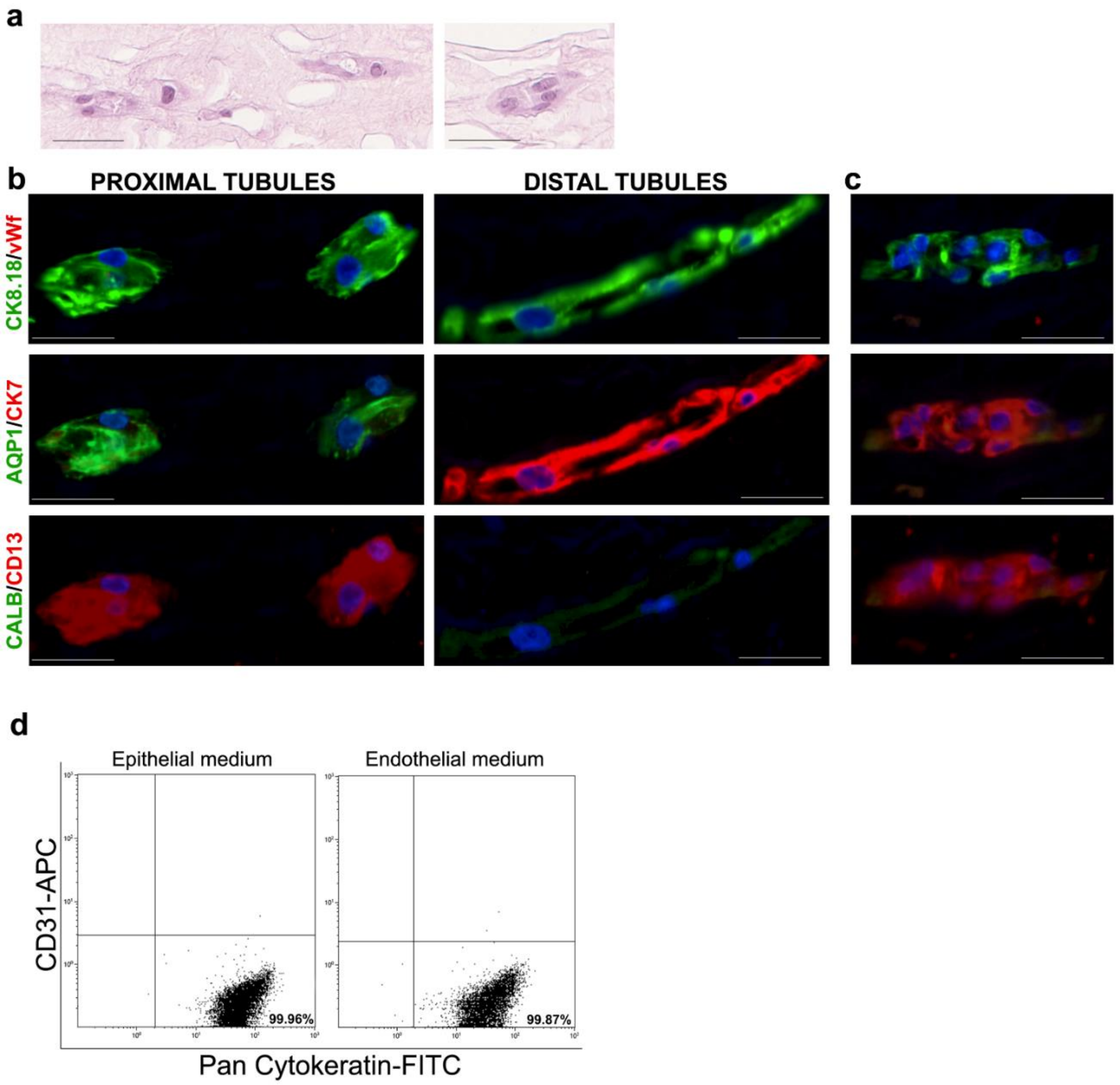

Supplementary Figure S2: Differentiation abilities of PKH$^{low/neg}$ progenitor cells. (a-b-c) Five independent experiments of human kidney scaffold repopulation for 30 days with PKH$^{low/neg}$ cells in presence of basal medium were performed. (**a**) Representative H&E staining of FFPE repopulated scaffold sections. Scale bars, 50 µm. (**b–c**) Representative sequential IF analysis of the FFPE repopulated scaffolds with the antibodies against the indicated markers that recognise specific tubular or vascular phenotypes. Scale bars, 50 µm. CK: Cytokeratin; AQP: Aquaporin; CALB: Calbindin D-28k; vWf: von Willebrand Factor; Blue:DAPI**.** (d) FACS analysis of representative clones obtained from single PKH$^{low/neg}$ cells at ten days of culture in epithelial and endothelial media. The CD31 and Cytokeratin markers were evaluated.

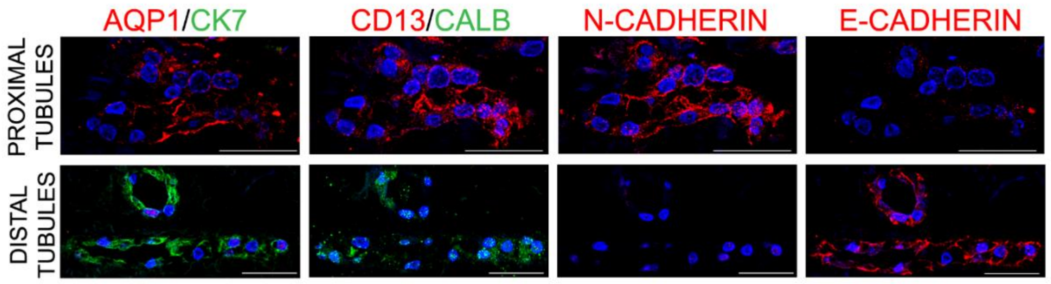

Supplementary Figure S3: Histological characterization of decellularized scaffolds repopulated with RSC. Representative sequential IF analysis of the FFPE repopulated scaffolds with the antibodies

against the indicated markers that recognise specific proximal (AQP1, CD13, N-Cadherin) or distal (CK7, CALB, E-Cadherin) tubular phenotypes. CK: Cytokeratin; AQP: Aquaporin; CALB: Calbindin D-28k.
